# Repurposing Old Drugs into New Epigenetic Inhibitors: Promising Candidates for Cancer Treatment?

**DOI:** 10.3390/pharmaceutics12050410

**Published:** 2020-04-29

**Authors:** Filipa Moreira-Silva, Vânia Camilo, Vítor Gaspar, João F. Mano, Rui Henrique, Carmen Jerónimo

**Affiliations:** 1 Cancer Biology and Epigenetics Group, IPO Porto Research Center (CI-IPOP), Portuguese Oncology Institute of Porto (IPO Porto), Rua Dr. António Bernardino de Almeida, 4200-072 Porto, Portugal; filipa.m.silva@ipoporto.min-saude.pt (F.M.-S.); vania.gomes.camilo@ipoporto.min-saude.pt (V.C.); 2 Department of Chemistry, CICECO, University of Aveiro, Campus Universitário de Santiago, 3810-193 Aveiro, Portugal; vm.gaspar@ua.pt (V.G.); jmano@ua.pt (J.F.M.); 3 Cancer Biology and Epigenetics Group, IPO Porto Research Center (CI-IPOP) and Department of Pathology, Portuguese Oncology Institute of Porto (IPO Porto), Rua Dr. António Bernardino de Almeida, 4200-072 Porto, Portugal; rmhenrique@icbas.up.pt

**Keywords:** cancer, prostate cancer, CRPC, drug repurposing, epigenetic, epi-drugs

## Abstract

Epigenetic alterations, as a cancer hallmark, are associated with cancer initiation, progression and aggressiveness. Considering, however, that these alterations are reversible, drugs that target epigenetic machinery may have an inhibitory effect upon cancer treatment. The traditional drug discovery pathway is time-consuming and expensive, and thus, new and more effective strategies are required. Drug Repurposing (DR) comprises the discovery of a new medical indication for a drug that is approved for another indication, which has been recalled, that was not accepted or failed to prove efficacy. DR presents several advantages, mainly reduced resources, absence of the initial target discovery process and the reduced time necessary for the drug to be commercially available. There are numerous old drugs that are under study as repurposed epigenetic inhibitors which have demonstrated promising results in in vitro tumor models. Herein, we summarize the DR process and explore several repurposed drugs with different epigenetic targets that constitute promising candidates for cancer treatment, highlighting their mechanisms of action.

## 1. Introduction

Cancer is a disease that affects millions of citizens worldwide [1], being the second most common cause of death after cardiovascular diseases.

**Figure 1:**
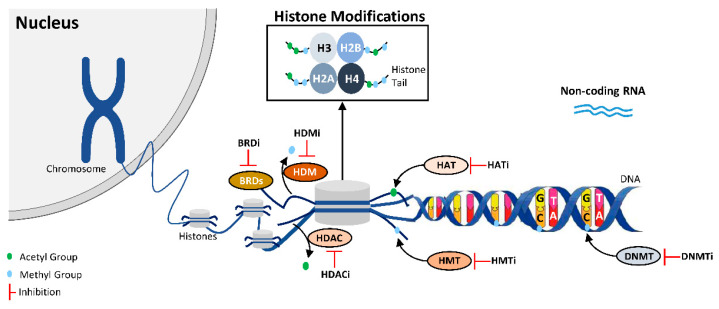
Epigenetic Mechanisms and Epigenetic Inhibitors. This figure illustrates the epigenetic enzymes responsible for DNA and Histone Modifications, along with illustrative inhibitors classified according their epigenetic target.

Both genetic and epigenetic mechanisms play an important role in malignant transformation, cancer initiation, tumor progression and prognosis [2]. Epigenetics comprises different modifications in gene expression patterns which do not derive from alterations in DNA sequence and that are reversible and heritable. The main epigenetic mechanisms described comprise DNA methylation, chromatin remodeling and microRNA regulation (Figure 1) [2]. Of all epigenetic mechanisms, DNA methylation is the most studied. The DNA methyltransferase enzymes (DNMTs) are responsible for the addition of a methyl group, donated by *S*-adenosylmethionine (SAM), to the fifth carbon of the cytosine of CpG dinucleotide [3]. Aberrant alteration of this mechanism, particularly DNA hypermethylation of regulatory regions of genes, is a common feature of cancer. Examples include *Glutathione S-transferase pi 1* (*GSTP1*), involved in DNA protection, androgen receptor (AR), in prostate cancer [4,5], estrogen receptor (ER) in breast cancer and adenomatous polyposis coli (APC) in colorectal cancer, among many others.

Alongside DNA methylation, histone modifications also play a role in carcinogenesis. Histones may endure posttranslational modifications at N-terminal tails, of which acetylation and methylation seem to be the most relevant [6]. Histone deacetylases (HDAC) are overexpressed in more advanced stages, for example HDAC1, HDAC2 and HDCA3 in castration-resistant prostate cancer (CRPC) [7]. Nonhistone proteins can undergo modifications by HDAC and histone acetyltransferases (HAT) [8]. Concerning histone methyltransferases (HMT), EZH2 is the most referred HMT, being responsible for the trimethylation of lysine 27 in histone 3 (H3K27me^3^) [9]. LSD1, a histone demethylase (HDM), is associated with aggressiveness and, in fact, it may form a complex with nonhistone proteins that promote cell proliferation and tumor progression [10].

Because epigenetic alterations are associated with cancer progression/aggressiveness, and considering that these alterations are reversible, drugs that target epigenetic enzymes may revert those alterations and contribute to the attenuation of the malignant phenotype (Figure 1).

## 2. Drug Repurposing

**Figure 2:**
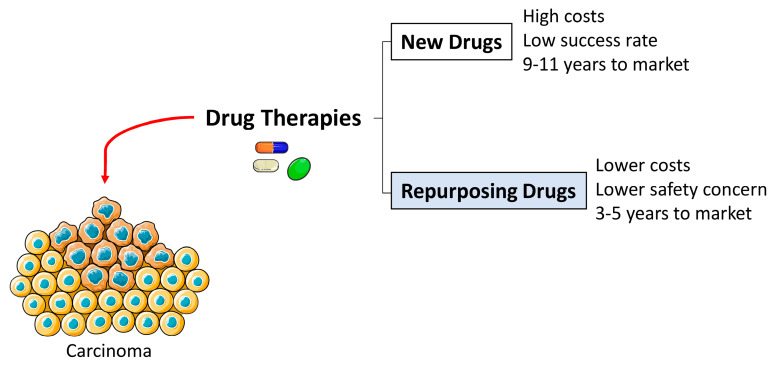
Drug Repurposing advantages over the discovery and development of new drugs for cancer treatment.

The traditional drug discovery pathway is costly, time consuming and has a low success rate. Considering these bottlenecks, new methodologies have been tested and the Drug Repurposing process has emerged as an interesting approach in cancer therapy (Figure 2).

Drug Repurposing (DR) refers to the process of discovery of a new medical indication for a drug that was approved for another indication, removed from the market due to adverse events, was not accepted for the proposed indication or failed to prove efficacy [11,12,13]. Different strategies may be used to identify potential repurposing drugs, specifically, network-based strategies which include cluster and propagation, text mining-based and semantic-based approaches [14]. DR presents several advantages compared to the traditional drug discovery pathway (Figure 2), i.e., mainly the reduced resources, absence of the initial target discovery process, previously assessed drug safety and the reduced time necessary for the drug to reach the market [13,14]. Nevertheless, DR also entails several challenges, including the choice of the right approach to investigate the repurposing potential of a drug. However, considerations involving the intellectual and economic property of the drug, the existence of available data regarding the compound structure, mechanism of action, efficacy and adverse events comprise the most challenging characteristics of this process [14].

There are several examples of drugs that were repurposed into new therapeutic approaches. Sildenafil is a drug that was originally developed for the treatment of coronary artery disease but which failed to pass on phase II clinical trials. One of the side effects verified upon treatment with Sildenafil was penile erection. Hence, this drug was repurposed by the FDA for the treatment of erectile dysfunction, in 1998 [11,14,15]. Other drugs have been repurposed for cancer therapy; one example is mebendazole, that was original indicated for the treatment of helminthic infections but was repurposed for the treatment of cancer, particularly, metastatic adrenocortical carcinoma and refractory metastatic colon cancer [14,16,17].

In Oncology, epigenetic alterations are becoming a therapeutic target. Food and Drug Administration (FDA) has approved two epigenetic modulators for cancer therapy that are repurposed drugs: 5-azacytidine and 5-aza-2′-deoxycytidine [14]. These drugs were approved due to their antimetabolic effects, but it was found that they were incorporated into DNA and inhibited DNA methylation [18,19,20]. Therefore, both were approved by the FDA for the treatment of myelodysplastic syndromes.

DR is an important tool for novel, targeted therapies, and in this review, we will explore several repurposing drugs for epigenetic targets that might be promising candidates for Prostate Cancer (PCa) treatment.

## 3. DNMT Inhibitors

DNMT inhibitors (DNMTi) are the most studied epigenetic inhibitors. Presently, there are several drugs that can be repurposed for DNMTi (Table 1).

Chlorogenic acid is a coffee polyphenol that has been shown to inhibit DNMT1. Its inhibitory effect is due to a chemical transformation resulting in increased formation of *S*-adenosyl-L-homocysteine (SAH) [21]. Using breast cancer cell lines, it was demonstrated that chlorogenic acid inhibits DNMT1, curbing DNA methylation [21]. In addition to in vitro models, the anticancer potential of this natural compound has been investigated in clinical trials involving patients with lung cancer (NCT03751592, recruiting), advanced solid tumors (NCT02245204, NCT02136342) and glioblastoma (NCT02728349). The natural compound harmine inhibited DNMT1 in acute myeloid leukemia cell lines through decreased DNMT1 gene expression, thus promoting *p15* promoter demethylation. It was also shown to have nonepigenetic effects, causing reduced cell proliferation and cell cycle arrest at G_0_/G_1_ phase [22,42]. Furthermore, laccaic acid was found to inhibit DNMT1 activity and promote the reactivation of genes silenced by promoter methylation in breast cancer cell lines [29] and in RGS6-/- mice [28]. Mahanine is a plant-derive alkaloid that inhibits DNMT1 and DNMT3B through proteasomal degradation [31]. In PCa cell lines, this compound inhibited DNMT activity, reducing *RASSF1A* promoter methylation and inducing re-expression [30,31].

The local anesthetic procaine is another interesting candidate for DR in cancer. It is a nonnucleoside inhibitor of DNMT1 and DNMT3A that binds to the binding pocket of the enzyme, disrupting the attachment of DNMTs to DNA [41]. In breast cancer cell lines, procaine induces DNA demethylation in CpG islands, triggering a 40% reduction in 5-methyl-cytosine (5mC) content and the re-expression of epigenetically-silenced genes [39]. In other tumor models, particularly gastric cancer, hepatocellular carcinoma (HCC) and nonsmall cell lung cancer (NSCLC), procaine also demonstrated nonepigenetic effects such as cell proliferation inhibition, apoptosis enhancement [41], cell cycle arrest [40] and downregulation of Wnt signaling pathway activation [37]. The FDA-approved drug procainamide is a derivative of procaine, used in the treatment of cardiac arrythmia. It was repurposed as a DNMT1 inhibitor. Procainamide interacts with the enzyme binding pocket and reduces the affinity of DNMT1 for hemimethylated DNA and SAM [38]. This drug inhibits DNMT1 activity, reverses CpG island methylation, decreasing 5mC content, and reduces gene-specific methylation at promoter sites [38]. In NSCLC, PCa, breast and bladder cancer, it induces the re-expression of methylated silenced genes, respectively, *WIF-1* [37], *GSTP1* [36], *ER*, *RARβ*, *p12* and *p16* [24]. Hydralazine is an arterial vasodilator approved by the FDA for the treatment of severe hypertension. It has been studied in recent years as a DNMTi in several tumor models. Hydralazine is a nonnucleoside DNMTi that interacts with the binding pocket of the enzyme with high affinity due to the presence of Lys162 and Arg24 [43,44]. Deng et al. [23] have shown that hydralazine can decrease DNMT1 and DNMT3A mRNA expression and protein levels in T cell leukemia cell lines. The effect of hydralazine in DNMT1 has also been studied in other tumor models. It was demonstrated that hydralazine induces DNA demethylation, decreases DNMT activity and promotes *RARβ*, *p21*, *p16* and *APC* gene expression in breast, bladder and cervical cancer cell lines, respectively [24,25,26]. Additionally, in cervical cancer cell models, this repurposed drug also showed nonepigenetic effects, particularly cell growth inhibition, cell cycle arrest at S phase and apoptosis enhancement [26]. In PCa, Graça et al. [27] showed that hydralazine decreases DNMT1 and also DNMT3A/3B mRNA expression, decreases DNMT1 protein levels, restores *AR* and *p21* expression and inhibits the Epidermal Growth Factor Receptor (EGFR) bypass signaling pathway [27]. Additionally, clinical trials are ongoing to investigate the demethylating potential of hydralazine in combination with HDACi valproic acid. This epigenetic combination is being tested in patients with several malignancies, including lung (NCT00996060), cervical (NCT00404326) and locally advanced breast (NCT00395655) cancers, as well as different solid tumors which are refractory to current therapies (NCT00404508). Moreover, the FDA-approved drug, olsalazine, a nucleoside DNMT inhibitor was first approved for the treatment of inflammatory bowel disease and ulcerative colitis, and later (2014) was shown to inhibit DNMT activity in cervical cancer cell lines [35].

Finally, some antibiotics are also being studied. Mithramycin A has the potential to inhibit DNMT. Lin et al. [32] studied the effect of mitramycin A in lung cancer cell lines and found that it decreases CpG island methylation, interacts with the catalytic pocket of DNMT1 inhibiting its activity, decreases DNMT1 protein levels and induces re-expression of silenced genes [32]. Nanaomycin A inhibits DNMT3B through molecular docking into the active site of the enzyme, which is stabilized by interaction with specific amino acids (Glu697, Arg731, Arg733) [33]. In liquid and solid tumors, nanaomycin A inhibits DNMT3B activity and reverses CpG methylation, thus reactivating silenced genes [33,34].

## 4. Inhibitors of Histone Modulators

### 4.1. HDAC Inhibitors

In PCa, HDAC enzymes are overexpressed, and due to the heterogeneity among subclasses, it is challenging to develop new drugs that target these epigenetic enzymes. Nonetheless, several approved drugs have been studied as potential HDAC inhibitors (HDACi) (Table 2).

Apicidin is a fungal metabolite that has been repurposed as an inhibitor of HDAC3, HDAC4 and HDAC8. Apicidin reduces HDAC3 and HDAC4 expression and activity, leading to increased histone H3 and H4 acetylation in endometrial and ovarian cancer cell lines [50,77]. In these models, it was also shown that apicidin has nonepigenetic effects, specifically, decreasing cell proliferation, enhancing apoptosis, and inhibiting migration and invasion [50,77]. In the ovarian cancer cell line SKOV-3, it inhibits HDAC4 binding to *Sp1* at *RECK* gene promoter [50]. Furthermore, it was also demonstrated that apicidin inhibits HDAC8, reducing its expression, increasing histone H4 acetylation, inhibiting cell growth and inducing apoptosis in an oral squamous cell carcinoma cell line [51]. In an in vivo mouse model, it was demonstrated that this repurposed drug inhibits tumor growth and decreases HDAC8 expression [51]. Moreover, apicidin also demonstrated an inhibitory effect on DNMT1 activity [46,47,78], and it was shown to be capable of inducing nonepigenetic effects, specifically, inhibition of cell proliferation, increase in apoptosis rate, upregulation of *p21* and *p27* expression, downregulation of *cyclin D1* and *cyclin E* gene expression and cell cycle arrest [45,48]. Furthermore, Pandey and colleagues [53] demonstrated that the natural compound aspigenin can be repurposed as an HDAC class I inhibitor. In PCa cell line PC-3, it was disclosed that this compound could inhibit class I HDACs, specifically HDAC1 and HDAC3 activity, inducing histone H3 and H4 acetylation, promoting cell cycle arrest and upregulating *p21* gene expression. Pandey and colleagues also verified the effect of aspigenin in an in vivo model, in which the compound reduced class I HDAC activity, decreasing HDAC1 and HDAC3 protein levels, reducing tumor size, promoting apoptosis and upregulating *p21* gene expression along with *Bcl-2* downregulation [53]. Ginseng is a plant extract extensively used in traditional Chinese Medicine that has been recently proposed as HDACi. In fact, in a NSCLC cell line, ginseng inhibited HDAC activity, upregulated *p21* gene expression and promoted cell death [57]. Furthermore, *Helminthosporium carbonum* (HC)-toxin, a cyclic tetrapeptide derived from a plant, has been identified as HDAC inhibitor in different cell models. In breast cancer cell lines, HC-toxin inhibits HDAC activity and promotes nonepigenetic effects, specifically, cell proliferation inhibition, cell death and cell cycle arrest at G_2_/M phase [58]. Additionally, in a neuroblastoma 2D cell culture model, HC-toxin inhibited HDAC activity and induced histone H4 acetylation [59]. The sponge *Pseudoceratina purpurea* derivative Psammaplin A can be reduced to its monomers inside the cells due to the presence of disulfide bounds. These monomers, which have thiol groups, are key factors for the inhibition HDAC activity [61]. Psammaplin A inhibits HDAC1 and HDAC6 activity, being more potent against HDAC1, reducing HDAC1 protein levels [61]. It also increases histone H3 and H4 acetylation and transcript levels [63,64]. In addition, this compound has also demonstrated nonepigenetic effects, in particular, cell and tumor growth inhibition [60], *p21* expression upregulation, inhibition of *Rb*, *cyclins* and *cyclin-dependent kinase* (*CDK)* gene expression, cell cycle arrest [63] and also stimulation of morphological changes [64]. Kim et al. [62] demonstrated that psammaplin A, at nanomolar levels, also inhibited sirtuin 1 (SIRT1) activity. In breast cancer cell lines, Kim and colleagues showed that this compound reduces SIRT1 enzymatic activity and protein levels, increases p51 acetylation, reduces nuclear levels of SIRT1 and discloses nonepigenetic effects, specifically, cell growth inhibition and cell cycle arrest at G_2_/M phase [62].

The antimalarial drug artemisin has been repurposed as an HDAC1, HDAC2 and HDAC6 inhibitor. In breast cancer cell line MCF-7, Kumari et al. [52] demonstrated that artemisin inhibits HDAC1, HDAC2 and HDAC6 activity, and displays nonepigenetic effects, including inhibition of cell proliferation, migration and invasion, and apoptosis enhancement [52]. Carbamazepine is an FDA-approved drug for the control of psychomotor or focal seizures, and, in recent years, it has been investigated as an HDACi. This drug inhibits HDAC activity and presents nonepigenetic effects, causing cell growth inhibition, increment of apoptosis rate and re-expression of silenced genes [56]. In breast cancer cell lines, carbamazepine inhibits HDAC6 activity, increases Hsp90 acetylation, induces HER2 protein degradation and upregulates *p21* gene expression [54]. On the other hand, in liver cancer cell lines, carbamazepine inhibits HDAC3 and HDAC7 and induces histone H4 acetylation [55]. Sodium butyrate is a short-chain fatty acid with anti-inflammatory proprieties that inhibits HDAC1. In solid tumor models (breast, prostate and gastric cancer), sodium butyrate increased histone H2B and H4 acetylation and demonstrated nonepigenetic effects, particularly, inhibition of cell proliferation, cell cycle arrest at G_1_/G_2_ phase and increased apoptosis [65,66,67]. Moreover, trichostatin A (TSA), an antifungal antibiotic with pan-HDACi activity is effective in several tumor models at the nanomolar level. TSA inhibits HDAC activity [69], downregulates HDAC1 expression [75], increases histone H4 [69] and estrogen receptor (ER) acetylation in breast cancer cell lines [76], increases histone H3 lysine 9 and lysine 27 acetylation [76] and upregulates *p21*, *p27* and *p57* expression in colon cancer cell lines [75] Additionally, in PCa, TSA increases histone H4 lysine 16 acetylation, particularly in CRPC cell lines [73], and affects p53 acetylation [49]. In addition, TSA presents nonepigenetic effects, including decreased cell proliferation [68,74], increased cell death [72,75] with an increase in active caspase-3 levels [71], increased hypoxic responses [74], downregulation of *cyclin D1* gene expression [71], cell cycle arrest at G_1_ phase, increased expression of *Bax* gene and downregulated *Bcl-2* gene expression and decreased phosphorylation of Akt and ERK proteins [70]. There is an ongoing clinical trial (NCT03838926) recruiting patients with hematological malignancies to investigate the anticancer effectiveness of TSA.

### 4.2. HAT, HMT, HDM and BET Inhibitors

In recent years, HAT, HMT, HDM and BET inhibitors have gained interest from the scientific community; several drugs have shown promise as repurposed inhibitors of these histone modulators (Table 3).

Several natural compounds have been investigated as repurposed histone modulators inhibitors. Anacardic acid, an extract from cashew nutshell, showed anticancer and anti-inflammatory properties. It was found to have HATi properties, specifically acting as a p300 and Tip60 inhibitor at nanomolar and micromolar levels [79]. Sun and colleagues demonstrated that anacardic acid inhibits Tip60 HAT activity, thus curbing ATM acetylation and sensitizing tumor cells to the cytotoxic effect of radiation [96]. Moreover, in several cell lines derived from liquid and solid tumors, anacardic acid inhibited p300 HAT activity, along with showing nonepigenetic effects, notably, the inhibition of IkBα and NF-kB activation, prevention of p65 acetylation and its nuclear translocation, potentiation of apoptosis via TNF-induced caspase activation and downregulation of the expression of several genes involved in invasion and angiogenesis [80]. Garcinol is a natural compound with antioxidant properties that showed promising results as a repurposed HATi, specifically, through p300 and KAT2B inhibition [82,85]. In several solid tumors, particularly, esophageal, hepatocellular, breast and cervical cancers, garcinol inhibits p300 levels and activity [85] alongside KAT2B inhibition [82], and reduces histone H3 lysine 18 acetylation [83]. On the other hand, garcinol depicts nonepigenetic effects, namely, cell cycle arrest, apoptosis enhancement, migration and invasion inhibition, decreased tumor cell proliferation [85], impaired angiogenesis [84] and inhibition of the activation of intracellular signaling pathways (e.g., TGFβ [85] and STAT3 [84]). In addition, plumbagin, a natural compound derived from *Plumbago zeylanica* has been repurposed as a noncompetitive p300 inhibitor. In a liver cancer cell line, plumbagin inhibited p300 HAT activity, hence preventing p53 acetylation; it also decreased histone H3 and H4 acetylation and showed nonepigenetic effects, specifically, enhancement of apoptosis [89]. Sakane et al. demonstrated that the natural compound geranylgeranoic acid (GGA) is a LSD1 inhibitor. In a neuroblastoma cell line, it was demonstrated that GGA increases histone H3 lysine 4 di-methylation, upregulates NTRK2 gene expression and inhibits tumor cells proliferation [86].

Furthermore, clorgyline and pargyline, both monoamine oxidase (MAO) inhibitors, have been repurposed as LSD1 inhibitors for solid and liquid tumors. In 2013, Han et al. investigated the effect of clorgyline in leukemia, colon and bladder cancer cell lines. They demonstrated that clorgyline inhibits LSD1 activity and decreases histone H3, lysine 4 mono and di-methylation, thus promoting an open chromatin state and the re-expression of silenced genes [81]. Additionally, in PCa cell line LNCaP, pargyline inhibits LSD1 activity and reduces histone H3 lysine 4 and lysine 9 di-methylation, as well as showing nonepigenetic effects, including pargyline-mediated upregulation of E-cadherin expression, along with downregulation of N-cadherin and vimentin expression, hence preventing epithelial-mesenchymal transition, migration and invasion [88].

Nitroxoline is an FDA-approved drug for the treatment of urinary infections which has been repurposed as BETi, with selectivity for BRD4. Nitroxoline occupies the acetylated lysine pocket of BRD4, preventing binding to acetylated lysine residues [87]. Therefore, in mixed-lineage leukemia cell lines, nitroxoline inhibited binding of BRD4 to acetylated histone H4 at a nanomolar level [87]. Ribavirin is an antiviral agent that blocks nuclei acid synthesis. It was approved by FDA for treatment of respiratory syncytial virus (RSV) infections and Hepatitis C. However, in recent years, ribavirin has gained interest as an HMTi, specifically, an EZH2 inhibitor. Ribavirin reduces EZH2 expression, at transcript and protein levels, as well as its activity [91,92], thereby preventing histone H3 lysine 27 trimethylation in numerous solid tumors [92]. Additionally, this antiviral agent possesses several nonepigenetic effects, including inhibition of tumor cell proliferation [92], downregulation of activation of several signaling pathways components (eIF4E, mTOR, ERK) [91], cell cycle arrest, increase in apoptosis, inhibition of migration and invasion. In an in vivo model, it reduced tumor growth and dissemination, improving the survival rate [90]. Different clinical trials have explored the potential of ribavirin for cancer treatment (NCT01056757, NCT01268579, NCT00559091). In a clinical trial involving patients with acute myeloid leukemia (NCT01056523), preliminary results showed that this compound was effective in reducing tumor cells growth, being well tolerated by patients. Moreover, tranylcypromine, a nonselective and irreversible MAO inhibitor approved by FDA for the treatment of depression, dysthymia, panic and phobia disorders, has been repurposed as LSD1 inhibitor. It has been shown that tranylcypromine inhibits LSD1 activity, reduces histone H3 lysine 4 di-methylation and increases histone H3 methylation [93,94,95]. The antineoplastic effect of tranylcypromine was investigated in clinical trials for leukemia (NCT02717884) and myelodysplastic syndrome (NCT02273102).

## 5. DNMT and HDAC Dual Inhibitors

Among epigenetic targets, DNMT and HDAC enzymes are the most studied. In cancer, altered DNMT and HDAC expression are linked together, driving downregulation of tumor suppressor gene expression [97]. Therefore, drugs that target both DNMT and HDAC enzymes could be an alternative approach to single target agents, with improved efficacy (Table 4).

Berberine is a natural compound used for the treatment of parasitic and fungal infections which has been repurposed as DNMT and HDAC dual inhibitor [109]. Regarding DNMT inhibition, in a multiple myeloma cell line, berberine downregulated DNMT1 and DNMT3A gene expression and activity, restoring *p53* expression through DNA hypomethylation [99]. Moreover, in a 2D lung cancer cell model, berberine showed strong inhibition of class I and II HDACs, downregulating gene expression and increasing histone H3 and H4 acetylation [100]. Additionally, berberine presents nonepigenetic effects: reduced cell proliferation, increased cell apoptosis, cell cycle arrest and inactivation of EGFR signaling pathway [98,99,100]. Parthenolide, also a natural compound, has anti-inflammatory properties and has been reported as HDACi and DNMTi in several tumor models. Indeed, parthenolide downregulates HDAC1 gene expression [104], induces HDAC1 proteosomal degradation, reducing its activity, and increases histone acetylation [101,102,110]. Moreover, this compound prevents Sp1 binding to DNMT1 promoter region, impairing its expression and activity, upregulates the expression of silenced genes and promotes a decrease in DNA methylation levels [103]. Additionally, parthenolide discloses nonepigenetic effects, including induction of apoptosis, cell cycle arrest, tumor growth inhibition and inactivation of several intracellular signaling pathways (NF-kB, STAT, MAPK) [101,103,104,110]. Furthermore, the natural compound resveratrol has been studied as HDACi and DNMTi. Resveratrol fits into the binding pocket of HDAC enzymes and, due to interaction with the zinc ion, inhibits HDAC activity [111]. In a breast cancer cell model, this compound inhibited HDAC and DNMT1 activity, decreasing histone H3 lysine 27 methylation and increasing its acetylation status [106,107]. Additionally, in a 2D thyroid cancer cell model, this compound downregulated DNMT gene expression and demethylated CpG sites at promoter regions [108]. Resveratrol enhances activating histone marks, reduces repressive histone marks [106] and induces gene promoter demethylation [105], upregulating the expression of silenced tumor suppressor genes (*BRCA1, p53, p21*). Moreover, the effect of resveratrol as a repurposed cancer drug was also investigated in clinical trials (NCT00256334, NCT01476592, NCT00433576).

## 6. Conclusions and Future Perspectives

The previously cited studies demonstrate that old drugs can be reused for new clinical applications, thus broadening their previously intended application (Supplementary Tables S1–S4). This is, indeed, a strength, since safety and pharmacokinetic profiles are already available, which fast-tracks their use in new clinical settings. Another advantage is that epigenetic mechanisms are shared across different tumour models, implying that their use can be widespread. Examples include DNMTi Hydralazine, Mahanine, Procainamide, HDACi TSA and Apicidin, and dual inhibitor Berberine. These compounds have been reported in the literature as being effective in different tumor models and, hence, seem to be the most promising compounds for further exploitation. Although the specific interactions between repurposed drugs and epigenetic enzymes are common to all tumour models, epigenetic inhibition effects might be diverse. Aberrant epigenetic mechanisms cause specific alterations in gene expression, cell cycle and proliferation according to tumour model, which might differentially impact on gene expression patterns.

However, most studies used a small range of drug concentrations in a limited number of cell lines, and mainly in 2D settings, thus failing to demonstrate efficacy in more complex models such as 3D culture and in vivo assays. In these 2D models, cells grow in synthetic plastic surfaces, which represents a highly reductionist model due to the loss of extracellular matrices (ECM), cell-cell communication, differentiation and polarization [112]. Therefore, more appropriate and complex cellular models are required to better represent human physiology and disease, such as 3D cell models. These in vitro models include 3D spheroids, organotypic cultures or organ-on-a-chip platforms [113]. The best model to be implemented for drug screening of solid human tumors seems to be 3D spheroids, which provide several features that mimic in vivo tissues such as 3D geometry, physical, chemical and biological gradients, cell stratification and functional differentiation [113]. Moreover, PCa development and progression are dependent on interactions between epithelial and stromal cells [114]. Consequently, stromal cells could influence the response of tumor epithelial cells to certain drugs and vice-versa. Therefore, 3D cell culture models that combine stromal and epithelial prostate cell lines (coculture models) are better suited to verify drug efficacy more accurately, because they represent an environment that is more closely related to an in vivo model. Thus, exploring the anticancer properties of repurposed drugs in those conditions seems to be a prerequisite before moving to in vivo models. Nevertheless, DR provides a novel framework for faster and, hopefully, less expensive development of therapies against the pervasive epigenetic alterations in human cancer which, until now, have mostly remained unexplored as effective therapeutic targets.

## Figures and Tables

**Table 1:** Noncancer drug repurposing candidates for DNMT inhibition.

Drug	Approved for	Epigenetic Target	Cancer Model
Chlorogenic Acid	Natural Compound (not approved)	DNMT1	Breast Cancer [21]
Harmine	Natural Compound (not approved)	DNMT1	Acute Myeloid Leukemia [22]
Hydralazine	Hypertension	DNMT1	T-Cell Leukemia [23], Breast Cancer [24,25], Bladder Cancer [24], Cervical Cancer [26], Prostate Cancer [27]
Laccaic Acid A	Natural Compound (not approved)	DNMT1	Breast Cancer [28,29]
Mahanine	Natural Compound (not approved)	DNMT1, DNMT3B	Prostate Cancer [30,31]
Mithramycin A	Hypercalcemia, especially due to malignancies	DNMT1	Lung Cancer [32]
Nanaomycin A	Quinone antibiotic (not approved)	DNMT3B	Lung Cancer, Colon Cancer [33], T-Cell Acute Lymphoblastic Leukemia, Burkitt Lymphoma [34]
Olsalazine	Inflammatory bowel disease and ulcerative colitis	DNMT	Cervical Cancer [35]
Procainamide	Cardiac arrythmias	DNMT1	Prostate Cancer [36], Breast Cancer, Bladder Cancer [37], Colon Cancer [38], Nonsmall Cell Lung Cancer [37]
Procaine	Infiltration anesthesia, peripheral nerve and spinal block	DNMT1, DNMT3A	Breast Cancer [39], Hepatocellular Carcinoma [40], Nonsmall Cell Lung Cancer [37], Gastric Cancer [41]

**Table 2:** Noncancer drug repurposing candidates for HDAC inhibition.

Drug	Approved for	Epigenetic Target	Cancer Model
Apicidin	Antiprotozoal (not approved)	HDAC3, HDAC4, HDAC8	Acute Promyelocytic Leukemia [45], Lung Cancer, Colon Cancer, Pancreatic Cancer [46], Cervical Cancer [47] Breast Cancer [48], Endometrial Cancer [49], Ovarian Cancer [50], Oral Squamous Cell Carcinoma [51]
Artemisin	Malaria	HDAC1, HDAC2, HDAC6	Breast Cancer [52]
Aspigenin	Natural Compound (not approved)	HDAC class I	Prostate Cancer [53]
Carbamazepine	Control of psychomotor or focal seizures	HDAC3, HDAC6, HDAC7	Breast Cancer [54], Liver Cancer [55], Colon Cancer [56]
Ginseng	Natural Compound (not approved)	HDAC	Nonsmall Cell Lung Cancer [57]
HC Toxin	Natural Compound (not approved)	HDAC	Breast Cancer [58], Neuroblastoma [59]
Psammaplin A	Natural Compound (not approved)	HDAC1, HDAC6, SIRT1	Lung Cancer [60], Breast Cancer [61,62], Endometrial Cancer [63], Cervical Cancer [64]
Sodium Butyrate	Anti-inflammatory	HDAC1	Gastric Cancer [65], Breast Cancer [66], Prostate Cancer [67]
TSA	Antifungal antibiotic	HDAC class I, II and SIRT6	Breast Cancer [68], Leukemia [69], Esophageal Squamous Carcinoma [70], Prostate Cancer [49,71,72,73], Pancreatic Cancer [74], Colon Cancer [75], Hepatocellular Carcinoma [76]

**Table 3:** Noncancer drug repurposing candidates for HAT, HMT, HDM and BET inhibition.

Drug	Approved for	Epigenetic Target	Cancer Model
Anarcadic Acid	Anti-inflammatory and radio-sensitization activities	Ep300 and Tip60	Cervical Cancer [79], Myeloid Leukemia, T-Cell Lymphoma, Lung Cancer, Prostate Cancer [80]
Clorgyline	MAO inhibitor	LSD1	Bladder Cancer, Colon Cancer, Leukemia [81]
Garcinol	Antioxidant (not approved)	Ep300 and KAT2B	Cervical Cancer [82], Breast Cancer [83], Hepatocellular Carcinoma [84], Esophageal Carcinoma [85]
Geranylgeranoic Acid	Natural Compound (not approved)	LSD1	Neuroblastoma [86]
Nitroxoline	Urinary antibacterial agent	BRD4	Mixed-Lineage Leukemia [87]
Pargyline	Irreversible selective MAO-B and antihypertensive	LSD1	Prostate Cancer [88]
Plumbagin	Natural Compound (not approved)	KAT3B/p300	Liver Carcinoma [89]
Ribavirin	RSV infections and Hepatitis C	EZH2	Solid Tumors [90,91,92]
Tranylcypromine	Depression, Dysthymic disorder, atypical depression, panic and phobic disorders	LSD1	Glioblastoma Multiforme [93], Sarcomas [94], Embryonal Carcinoma [95]

**Table 4:** Noncancer drug repurposing candidates for dual inhibition of DNMT and HDAC.

Drug	Approved for	Epigenetic Target	Cancer Model
Berberine	Parasitic and fungal infections	HDAC class I, II, IV and DNMT1, DNMT3A	Prostate Cancer [98], Multiple Myeloma [99], Lung Cancer [100]
Parthenolide	Anti-inflammatory (not approved)	HDAC1 and DNMT	Breast Cancer [101,102], Leukemia [103], Myeloma [104], Colon Cancer [102]
Resveratrol	Natural Compound (not approved)	HDAC and DNMT1	Nonsmall Cell Lung Cancer [105], Breast Cancer [106,107], Thyroid Cancer [108]

## Supplementary Materials

The following are available online at https://www.mdpi.com/1999-4923/12/5/410/s1; Table S1: Description of several noncancer drug repurposing candidates for DNMT inhibition; Table S2: Description of several noncancer drug repurposing candidates for HDAC inhibition; Table S3: Description of several noncancer drug repurposing candidates for HAT, HMT, HDM and BET inhibition; Table S4: Description of several noncancer drug repurposing candidates for DNMT and HDAC dual inhibition.

## References

[1] Bray F., Ferlay J., Soerjomataram I., Siegel R.L., Torre L.A., Jemal A. (2018). Global cancer statistics 2018: GLOBOCAN estimates of incidence and mortality worldwide for 36 cancers in 185 countries. CA A Cancer J. Clin..

[2] Jerónimo C., Bastian P.J., Bjartell A., Carbone G.M., Catto J.W., Clark S.J., Henrique R., Nelson W.G., Shariat S.F. (2011). Epigenetics in Prostate Cancer: Biologic and Clinical Relevance. Eur. Urol..

[3] Jurkowska R.Z., Jurkowski T., Jeltsch A. (2010). Structure and Function of Mammalian DNA Methyltransferases. ChemBioChem.

[4] Millar D.S., Ow K.K., Paul C.L., Russell P.J., Molloy P.L., Clark S.J. (1999). Detailed methylation analysis of the glutathione S-transferase pi (GSTP1) gene in prostate cancer. Oncogene.

[5] Schayek H., Bentov I., Sun S., Plymate S.R., Werner H. (2010). Progression to metastatic stage in a cellular model of prostate cancer is associated with methylation of the androgen receptor gene and transcriptional suppression of the insulin-like growth factor-I receptor gene. Exp. Cell Res..

[6] Kouzarides T. (2007). Chromatin Modifications and Their Function. Cell.

[7] Weichert W., Röske A., Gekeler V., Beckers T., Stephan C., Jung K., Fritzsche F.R., Niesporek S., Denkert C., Dietel M. (2008). Histone deacetylases 1, 2 and 3 are highly expressed in prostate cancer and HDAC2 expression is associated with shorter PSA relapse time after radical prostatectomy. Br. J. Cancer.

[8] Novotny-Diermayr V., Sangthongpitag K., Hu C.Y., Wu X., Sausgruber N., Yeo P., Greicius G., Pettersson S., Liang A.L., Loh Y.K. (2010). SB939, a Novel Potent and Orally Active Histone Deacetylase Inhibitor with High Tumor Exposure and Efficacy in Mouse Models of Colorectal Cancer. Mol. Cancer Ther..

[9] Varambally S., Dhanasekaran S.M., Zhou M., Barrette T.R., Kumar-Sinha C., Sanda M.G., Ghosh D., Pienta K.J., Sewalt R.G.A.B., Otte A.P. (2002). The polycomb group protein EZH2 is involved in progression of prostate cancer. Nature.

[10] Metzger E., Wissmann M., Yin N., Müller J.M., Schneider R., Peters A.H.F.M., Günther T., Buettner R., Schüle R. (2005). LSD1 demethylates repressive histone marks to promote androgen-receptor-dependent transcription. Nature.

[11] Ashburn T.T., Thor K.B. (2004). Drug repositioning: Identifying and developing new uses for existing drugs. Nat. Rev. Drug Discov..

[12] Peyvandipour A., Saberian N., Shafi A., Donato M., Draghici S. (2018). A novel computational approach for drug repurposing using systems biology. Bioinformatics.

[13] Shim J.S., Liu J.O. (2014). Recent Advances in Drug Repositioning for the Discovery of New Anticancer Drugs. Int. J. Boil. Sci..

[14] Naveja J.J., Dueñas-González A., Medina-Franco J.L., Medina-Franco J.L. (2016). Chapter 12—Drug Repurposing for Epigenetic Targets Guided by Computational Methods. Epi-Informatics.

[15] Li Y.Y., Jones S.J.M. (2012). Drug repositioning for personalized medicine. Genome Med..

[16] Pantziarka P., Bouche G., Meheus L., Sukhatme V., Sukhatme V.P., Vikas P. (2014). The Repurposing Drugs in Oncology (ReDO) Project. Ecancermedicalscience.

[17] Nygren P., Larsson R. (2013). Drug repositioning from bench to bedside: Tumour remission by the antihelmintic drug mebendazole in refractory metastatic colon cancer. Acta Oncol..

[18] Christman J.K. (2002). 5-Azacytidine and 5-aza-2′-deoxycytidine as inhibitors of DNA methylation: Mechanistic studies and their implications for cancer therapy. Oncogene.

[19] Fenaux P., Mufti G.J., Hellström-Lindberg E., Santini V., Finelli C., Giagounidis A., Schoch R., Gattermann N., Sanz G., List A.F. (2009). Efficacy of azacitidine compared with that of conventional care regimens in the treatment of higher-risk myelodysplastic syndromes: A randomised, open-label, phase III study. Lancet Oncol..

[20] Kantarjian H., Issa J.-P., Rosenfeld C.S., Bennett J.M., Albitar M., DiPersio J., Klimek V., Slack J., De Castro C., Ravandi F. (2006). Decitabine improves patient outcomes in myelodysplastic syndromes. Cancer.

[21] Lee W.J., Zhu B.T. (2005). Inhibition of DNA methylation by caffeic acid and chlorogenic acid, two common catechol-containing coffee polyphenols. Carcinogenesis.

[22] Oodi A., Norouzi H., Amirizadeh N., Nikougoftar M., Vafaie Z. (2017). Harmine, a Novel DNA Methyltransferase 1 Inhibitor in the Leukemia Cell Line. Indian J. Hematol. Blood Transfus..

[23] Deng C., Lu Q., Zhang Z., Rao T., Attwood J., Yung R., Richardson B. (2003). Hydralazine may induce autoimmunity by inhibiting extracellular signal-regulated kinase pathway signaling. Arthritis Rheum..

[24] Segura-Pacheco B., Trejo-Becerril C., Perez-Cardenas E., Taja L., Mariscal I., Chavez A., Acuña C., Salazar A.M., Lizano M., Dueñas-Gonzalez A. (2003). Reactivation of tumor suppressor genes by the cardiovascular drugs hydralazine and procainamide and their potential use in cancer therapy. Clin. Cancer Res..

[25] Segura-Pacheco B., Perez-Cardenas E., Taja-Chayeb L., Chávez-Blanco A.D., Vázquez A.L.R., Bribiesca L.B., Duenas-Gonzalez A. (2006). Global DNA hypermethylation-associated cancer chemotherapy resistance and its reversion with the demethylating agent hydralazine. J. Transl. Med..

[26] Song Y., Zhang C. (2008). Hydralazine inhibits human cervical cancer cell growth in vitro in association with APC demethylation and re-expression. Cancer Chemother. Pharmacol..

[27] Graça M.I.P.D.S., Sousa E.J., Costa-Pinheiro P., Vieira A.F.Q., Torres-Ferreira J., Martins M.G., Henrique R., Jerónimo C. (2014). Anti-neoplastic properties of hydralazine in prostate cancer. Oncotarget.

[28] Huang J., Stewart A., Maity B., Hagen J., Fagan R.L., Yang J., Quelle D.E., Brenner C., Fisher R.A. (2013). RGS6 suppresses Ras-induced cellular transformation by facilitating Tip60-mediated Dnmt1 degradation and promoting apoptosis. Oncogene.

[29] Fagan R.L., Cryderman D.E., Kopelovich L., Wallrath L., Brenner C. (2013). Laccaic Acid A Is a Direct, DNA-competitive Inhibitor of DNA Methyltransferase 1*. J. Boil. Chem..

[30] Jagadeesh S., Sinha S., Pal B.C., Bhattacharya S., Banerjee P.P. (2007). Mahanine reverses an epigenetically silenced tumor suppressor gene RASSF1A in human prostate cancer cells. Biochem. Biophys. Res. Commun..

[31] Agarwal S., Amin K.S., Jagadeesh S., Baishya G., Rao P.G., Barua N.C., Bhattacharya S., Banerjee P.P. (2013). Mahanine restores RASSF1A expression by down-regulating DNMT1 and DNMT3B in prostate cancer cells. Mol. Cancer.

[32] Lin R.-K., Hsu C.-H., Wang Y.-C. (2007). Mithramycin A inhibits DNA methyltransferase and metastasis potential of lung cancer cells. Anti-Cancer Drugs.

[33] Kuck D., Caulfield T., Lyko F., Medina-Franco J.L. (2010). Nanaomycin A Selectively Inhibits DNMT3B and Reactivates Silenced Tumor Suppressor Genes in Human Cancer Cells. Mol. Cancer Ther..

[34] Poole C.J., Zheng W., Lodh A., Yevtodiyenko A., Liefwalker D., Li H., Felsher D.W., Van Riggelen J. (2017). DNMT3B overexpression contributes to aberrant DNA methylation and MYC-driven tumor maintenance in T-ALL and Burkitt’s lymphoma. Oncotarget.

[35] Méndez-Lucio O., Tran J., Medina-Franco J.L., Meurice N., Muller M. (2014). Toward Drug Repurposing in Epigenetics: Olsalazine as a Hypomethylating Compound Active in a Cellular Context. ChemMedChem.

[36] Lin X., Asgari K., Putzi M.J., Gage W.R., Yu X., Cornblatt B.S., Kumar A., Piantadosi S., Deweese T.L., De Marzo A.M. (2001). Reversal of GSTP1 CpG island hypermethylation and reactivation of pi-class glutathione S-transferase (GSTP1) expression in human prostate cancer cells by treatment with procainamide. Cancer Res..

[37] Jablons D., Gao Z., Xu Z., Hung M.-S., Lin Y.-C., Wang T., Gong M., Zhi X., You L. (2009). Procaine and procainamide inhibit the Wnt canonical pathway by promoter demethylation of WIF-1 in lung cancer cells. Oncol. Rep..

[38] Lee B.H., Yegnasubramanian S., Lin X., Nelson W. (2005). Procainamide Is a Specific Inhibitor of DNA Methyltransferase 1. J. Boil. Chem..

[39] Villar-Garea A., Fraga M.F., Espada J., Esteller M. (2003). Procaine is a DNA-demethylating agent with growth-inhibitory effects in human cancer cells. Cancer Res..

[40] Tada M., Imazeki F., Fukai K., Sakamoto A., Arai M., Mikata R., Tokuhisa T., Yokosuka O. (2007). Procaine inhibits the proliferation and DNA methylation in human hepatoma cells. Hepatol. Int..

[41] Li Y.-C., Wang Y., Li D.-D., Zhang Y., Li C.-F., Zhao T.-C. (2017). Procaine is a specific DNA methylation inhibitor with anti-tumor effect for human gastric cancer. J. Cell. Biochem..

[42] Wong K.K., Lawrie C.H., Green T.M. (2019). Oncogenic Roles and Inhibitors of DNMT1, DNMT3A, and DNMT3B in Acute Myeloid Leukaemia. Biomark. Insights.

[43] Arce C., Segura-Pacheco B., Perez-Cardenas E., Taja-Chayeb L., Candelaria M., Duenas-Gonzalez A. (2006). Hydralazine target: From blood vessels to the epigenome. J. Transl. Med..

[44] Angeles E., HugoVazquez-Valadez V., Vazquez-Valadez O., Velazquez-Sanchez A., Ramirez A., Martínez L., Díaz-Barriga S., Romero-Rojas A., Cabrera G., Lopez-Castañares R. (2005). Computational Studies of 1-Hydrazinophthalazine (Hydralazine) as Antineoplasic Agent. Docking Studies on Methyltransferase. Lett. Drug Des. Discov..

[45] Hong J., Ishihara K., Yamaki K., Hiraizumi K., Ohno T., Ahn J.W., Zee O., Ohuchi K. (2003). Apicidin, a histone deacetylase inhibitor, induces differentiation of HL-60 cells. Cancer Lett..

[46] Wu L.-P., Wang X., Li L., Zhao Y., Lu S., Yu Y., Zhou W., Liu X., Yang J., Zheng Z. (2008). Histone Deacetylase Inhibitor Depsipeptide Activates Silenced Genes through Decreasing both CpG and H3K9 Methylation on the Promoter. Mol. Cell. Boil..

[47] Durczak M., Jagodzinski P.P. (2010). Apicidin upregulates PHD2 prolyl hydroxylase gene expression in cervical cancer cells. Anti-Cancer Drugs.

[48] Im J.Y., Park H., Kang K.W., Choi W.S., Kim H.S. (2008). Modulation of cell cycles and apoptosis by apicidin in estrogen receptor (ER)-positive and-negative human breast cancer cells. Chem. Interact..

[49] Reddy E.S.P., Fortson W.S., Kayarthodi S., Fujimura Y., Xu H., Matthews R., Grizzle W.E., Rao V.N., Bhat G.K. (2011). Histone deacetylase inhibitors, valproic acid and trichostatin-A induce apoptosis and affect acetylation status of p53 in ERG-positive prostate cancer cells. Int. J. Oncol..

[50] Ahn M.Y., Kang D.O., Na Y.J., Yoon S., Choi W.S., Kang K.W., Chung H.Y., Jung J.H., Min S., Kim H.S. (2012). Histone deacetylase inhibitor, apicidin, inhibits human ovarian cancer cell migration via class II histone deacetylase 4 silencing. Cancer Lett..

[51] Ahn M. (2018). HDAC inhibitor apicidin suppresses murine oral squamous cell carcinoma cell growth in vitro and in vivo via inhibiting HDAC8 expression. Oncol. Lett..

[52] Kumari K., Keshari S., Sengupta D., Sabat S.C., Mishra S.K. (2017). Transcriptome analysis of genes associated with breast cancer cell motility in response to Artemisinin treatment. BMC Cancer.

[53] Pandey M., Kaur P., Shukla S., Abbas A., Fu P., Gupta S. (2011). Plant flavone apigenin inhibits HDAC and remodels chromatin to induce growth arrest and apoptosis in human prostate cancer cells: In vitro and in vivo study. Mol. Carcinog..

[54] Meng Q., Chen X., Sun L., Zhao C., Sui G., Cai L. (2010). Carbamazepine promotes Her-2 protein degradation in breast cancer cells by modulating HDAC6 activity and acetylation of Hsp90. Mol. Cell. Biochem..

[55] Beutler A., Li S., Nicol R., Walsh M.J. (2005). Carbamazepine is an inhibitor of histone deacetylases. Life Sci..

[56] Akbarzadeh L., Zanjani T.M., Sabetkasaei M. (2016). Comparison of Anticancer Effects of Carbamazepine and Valproic Acid. Iran. Red Crescent Med. J..

[57] Byun M.R., Lee D.H., Jang Y.P., Lee H.S., Choi J.W., Lee S. (2019). Repurposing natural products as novel HDAC inhibitors by comparative analysis of gene expression profiles. Phytomedicine.

[58] Joung K.E., Kim D.-K., Sheen Y. (2004). Antiproliferative effect of trichostatin A and HC-toxin in T47D human breast cancer cells. Arch. Pharmacal Res..

[59] Deubzer H.E., Ehemann V., Westermann F., Heinrich R., Mechtersheimer G., Kulozik A.E., Schwab M., Witt O. (2007). Histone deacetylase inhibitor Helminthosporium carbonum (HC)-toxin suppresses the malignant phenotype of neuroblastoma cells. Int. J. Cancer.

[60] Pina I.C., Gautschi J.T., Wang G.-Y.-S., Sanders M.L., Schmitz F.J., France D., Cornell-Kennon S., Sambucetti L.C., Remiszewski S.W., Perez L.B. (2003). Psammaplins from the SpongePseudoceratinapurpurea:Inhibition of Both Histone Deacetylase and DNA Methyltransferase. J. Org. Chem..

[61] Baud M., Leiser T., Haus P., Samlal S., Wong A.C., Wood R.J., Petrucci V., Gunaratnam M., Hughes S.M., Buluwela L. (2012). Defining the Mechanism of Action and Enzymatic Selectivity of Psammaplin A against Its Epigenetic Targets. J. Med. Chem..

[62] Kim T.H., Kim H.S., Kang Y.J., Yoon S., Lee J., Choi W.S., Jung J.H., Kim H.S. (2015). Psammaplin A induces Sirtuin 1-dependent autophagic cell death in doxorubicin-resistant MCF-7/adr human breast cancer cells and xenografts. Biochim. Biophys. Acta.

[63] Ahn M.Y., Jung J.H., Na Y.J., Kim H.S. (2008). A natural histone deacetylase inhibitor, Psammaplin A, induces cell cycle arrest and apoptosis in human endometrial cancer cells. Gynecol. Oncol..

[64] Kim D.H., Shin J., Kwon H.J. (2007). Psammaplin A is a natural prodrug that inhibits class I histone deacetylase. Exp. Mol. Med..

[65] Shin H., Lee Y.S., Lee Y.C. (2011). Sodium butyrate-induced DAPK-mediated apoptosis in human gastric cancer cells. Oncol. Rep..

[66] Li L., Sun Y., Liu J., Wu X., Chen L., Ma L., Wu P. (2015). Histone deacetylase inhibitor sodium butyrate suppresses DNA double strand break repair induced by etoposide more effectively in MCF-7 cells than in HEK293 cells. BMC Biochem..

[67] Cang S., Xu X., Ma Y., Liu D., Chiao J.W. (2016). Hypoacetylation, hypomethylation, and dephosphorylation of H2B histones and excessive histone deacetylase activity in DU-145 prostate cancer cells. J. Hematol. Oncol..

[68] Vigushin D.M., Ali S., Pace P.E., Mirsaidi N., Ito K., Adcock I., Coombes R.C. (2001). Trichostatin A is a histone deacetylase inhibitor with potent antitumor activity against breast cancer in vivo. Clin. Cancer Res..

[69] Chambers A., Banerjee S., Chaplin T., Dunne J., Debernardi S., Joel S., Young B. (2003). Histone acetylation-mediated regulation of genes in leukaemic cells. Eur. J. Cancer.

[70] Ma J., Guo X., Zhang S., Liu H., Lu J., Dong Z., Liu K., Ming L. (2015). Trichostatin A, a histone deacetylase inhibitor, suppresses proliferation and promotes apoptosis of esophageal squamous cell lines. Mol. Med. Rep..

[71] Motawi T., Darwish H.A., Diab I., Helmy M.W., Noureldin M.H. (2018). Combinatorial strategy of epigenetic and hormonal therapies: A novel promising approach for treating advanced prostate cancer. Life Sci..

[72] Zhang H., Zhao X., Liu H., Jin H., Ji Y. (2019). Trichostatin A inhibits proliferation of PC3 prostate cancer cells by disrupting the EGFR pathway. Oncol. Lett..

[73] Xu Q., Liu X., Zhu S., Hu X., Niu H., Zhang X., Zhu D., Nesa E.U., Tian K., Yuan H. (2018). Hyper-acetylation contributes to the sensitivity of chemo-resistant prostate cancer cells to histone deacetylase inhibitor Trichostatin A. J. Cell. Mol. Med..

[74] Tiffon C. (2018). Histone Deacetylase Inhibition Restores Expression of Hypoxia-Inducible Protein NDRG1 in Pancreatic Cancer. Pancreas.

[75] Kavoosi F., Sanaei M. (2019). Effect of 5-aza-2′-deoxycytidine in comparison to valproic acid and trichostatin a on histone deacetylase 1, dna methyltransferase 1, and cip/kip family (p21, p27, and p57) genes expression, cell growth inhibition, and apoptosis induction in colon cancer sw480 cell line. Adv. Biomed. Res..

[76] Hernández-Oliveras A., Izquierdo-Torres E., Meneses-Morales I., Rodríguez G., Zarain-Herzberg A., Santiago-García J. (2019). Histone deacetylase inhibitors promote ATP2A3 gene expression in hepatocellular carcinoma cells: p300 as a transcriptional regulator. Int. J. Biochem. Cell Boil..

[77] Ahn M., Chung H.Y., Choi W.S., Lee B.-M., Yoon S., Kim H.S. (2010). Anti-tumor effect of apicidin on Ishikawa human endometrial cancer cells both in vitro and in vivo by blocking histone deacetylase 3 and 4. Int. J. Oncol..

[78] You J.S., Kang J., Lee E.K., Lee J.C., Lee S.H., Jeon Y.J., Koh D.H., Ahn S.H., Seo D.-W., Lee H.Y. (2007). Histone deacetylase inhibitor apicidin downregulates DNA methyltransferase 1 expression and induces repressive histone modifications via recruitment of corepressor complex to promoter region in human cervix cancer cells. Oncogene.

[79] Balasubramanyam K., Swaminathan V., Ranganathan A., Kundu T.K. (2003). Small Molecule Modulators of Histone Acetyltransferase p300. J. Boil. Chem..

[80] Sung B., Pandey M.K., Ahn K.S., Yi T., Chaturvedi M.M., Liu M., Aggarwal B.B. (2008). Anacardic acid (6-nonadecyl salicylic acid), an inhibitor of histone acetyltransferase, suppresses expression of nuclear factor-kappaB-regulated gene products involved in cell survival, proliferation, invasion, and inflammation through inhibition of the inhibitory subunit of nuclear factor-kappaBalpha kinase, leading to potentiation of apoptosis. Blood.

[81] Han H., Yang X., Pandiyan K., Liang G. (2013). Synergistic Re-Activation of Epigenetically Silenced Genes by Combinatorial Inhibition of DNMTs and LSD1 in Cancer Cells. PLoS ONE.

[82] Balasubramanyam K., Altaf M., Varier R.A., Swaminathan V., Ravindran A., Sadhale P.P., Kundu T.K. (2004). Polyisoprenylated Benzophenone, Garcinol, a Natural Histone Acetyltransferase Inhibitor, Represses Chromatin Transcription and Alters Global Gene Expression. J. Boil. Chem..

[83] Collins H., Abdelghany M.K., Messmer M., Yue B., Deeves S.E., Kindle K.B., Mantelingu K., Aslam A., Winkler S., Kundu T.K. (2013). Differential effects of garcinol and curcumin on histone and p53 modifications in tumour cells. BMC Cancer.

[84] Sethi G., Chatterjee S., Rajendran P., Li F., Shanmugam M.K., Wong K.-F., Kumar A.P., Senapati P., Behera A.K., Hui K.M. (2014). Inhibition of STAT3 dimerization and acetylation by garcinol suppresses the growth of human hepatocellular carcinoma in vitro and in vivo. Mol. Cancer.

[85] Wang J., Wu M., Zheng D., Zhang H., Lv Y., Zhang L., Tan H.S., Zhou H., Lao Y.Z., Xu H.X. (2019). Garcinol inhibits esophageal cancer metastasis by suppressing the p300 and TGF-beta1 signaling pathways. Acta Pharmacol. Sin..

[86] Sakane C., Okitsu T., Wada A., Sagami H., Shidoji Y. (2014). Inhibition of lysine-specific demethylase 1 by the acyclic diterpenoid geranylgeranoic acid and its derivatives. Biochem. Biophys. Res. Commun..

[87] Jiang H., Xing J., Wang C., Zhang H., Yue L., Wan X., Chen W., Ding H., Xie Y., Tao H. (2017). Discovery of novel BET inhibitors by drug repurposing of nitroxoline and its analogues. Org. Biomol. Chem..

[88] Wang M., Liu X., Guo J., Weng X., Jiang G., Wang Z., He L. (2015). Inhibition of LSD1 by Pargyline inhibited process of EMT and delayed progression of prostate cancer in vivo. Biochem. Biophys. Res. Commun..

[89] Ravindra K.C., Selvi B.R., Arif M., Reddy B.A.A., Thanuja G.R., Agrawal S., Pradhan S.K., Nagashayana N., Dasgupta D., Kundu T.K. (2009). Inhibition of Lysine Acetyltransferase KAT3B/p300 Activity by a Naturally Occurring Hydroxynaphthoquinone, Plumbagin*. J. Boil. Chem..

[90] Casaos J., Huq S., Lott T., Felder R., Choi J., Gorelick N., Peters M., Xia Y., Maxwell R., Zhao T. (2018). Ribavirin as a potential therapeutic for atypical teratoid/rhabdoid tumors. Oncotarget.

[91] Chen J., Xu X., Chen J. (2018). Clinically relevant concentration of anti-viral drug ribavirin selectively targets pediatric osteosarcoma and increases chemosensitivity. Biochem. Biophys. Res. Commun..

[92] De La Cruz-Hernández E., Medina-Franco J.L., Trujillo J., Chávez-Blanco A.D., Dominguez-Gomez G., Perez-Cardenas E., Gonzalez-Fierro A., Taja L., Dueñas-Gonzalez A. (2015). Ribavirin as a tri-targeted antitumor repositioned drug. Oncol. Rep..

[93] Singh M.M., Manton C., Bhat K.P., Tsai W.-W., Aldape K., Barton S., Chandra J. (2011). Inhibition of LSD1 sensitizes glioblastoma cells to histone deacetylase inhibitors. Neuro-Oncology.

[94] Bennani-Baiti I.M., Machado I., Llombart-Bosch A., Kovar H. (2012). Lysine-specific demethylase 1 (LSD1/KDM1A/AOF2/BHC110) is expressed and is an epigenetic drug target in chondrosarcoma, Ewing’s sarcoma, osteosarcoma, and rhabdomyosarcoma. Hum. Pathol..

[95] Lee M.G., Wynder C., Schmidt D.M., McCafferty D.G., Shiekhattar R. (2006). Histone H3 Lysine 4 Demethylation Is a Target of Nonselective Antidepressive Medications. Chem. Boil..

[96] Sun Y., Jiang X., Chen S., Price B.D. (2006). Inhibition of histone acetyltransferase activity by anacardic acid sensitizes tumor cells to ionizing radiation. FEBS Lett..

[97] Yuan Z., Chen S., Gao C., Dai Q., Zhang C., Sun Q., Lin J.-S., Guo C., Chen Y.Z., Jiang Y. (2019). Development of a versatile DNMT and HDAC inhibitor C02S modulating multiple cancer hallmarks for breast cancer therapy. Bioorganic Chem..

[98] Huang Z.-H., Zheng H.-F., Wang W.-L., Wang Y., Zhong L.-F., Wu J.-L., Li Q.-X. (2014). Berberine targets epidermal growth factor receptor signaling to suppress prostate cancer proliferation in vitro. Mol. Med. Rep..

[99] Qing Y., Hu H., Liu Y., Feng T., Meng W., Jiang L., Sun Y., Yao Y. (2014). Berberine induces apoptosis in human multiple myeloma cell line U266 through hypomethylation of p53 promoter. Cell Boil. Int..

[100] Kalaiarasi A., Anusha C., Sankar R., Rajasekaran S., Marshal J.J., Muthusamy K., Ravikumar V. (2016). Plant Isoquinoline Alkaloid Berberine Exhibits Chromatin Remodeling by Modulation of Histone Deacetylase To Induce Growth Arrest and Apoptosis in the A549 Cell Line. J. Agric. Food Chem..

[101] Gopal Y.V., Arora T.S., Van Dyke M.W. (2007). Parthenolide Specifically Depletes Histone Deacetylase 1 Protein and Induces Cell Death through Ataxia Telangiectasia Mutated. Chem. Boil..

[102] Dawood M., Ooko E., Efferth T. (2019). Collateral Sensitivity of Parthenolide via NF-κB and HIF-α Inhibition and Epigenetic Changes in Drug-Resistant Cancer Cell Lines. Front. Pharmacol..

[103] Liu Z., Liu S., Xie Z., Pavlovicz R.E., Wu J., Chen P., Aimiuwu J., Pang J., Bhasin D., Neviani P. (2009). Modulation of DNA methylation by a sesquiterpene lactone parthenolide. J. Pharmacol. Exp. Ther..

[104] Hartman M.L., Talar B., Sztiller-Sikorska M., Nejc D., Czyz M. (2016). Parthenolide induces MITF-M downregulation and senescence in patient-derived MITF-M(high) melanoma cell populations. Oncotarget.

[105] Fudhaili A., Yoon N.A., Kang S., Ryu J., Jeong J.Y., Lee N.H., Kang S.S. (2018). Resveratrol epigenetically regulates the expression of zinc finger protein 36 in nonsmall cell lung cancer cell lines. Oncol. Rep..

[106] Chatterjee B., Ghosh K., Kanade S.R. (2019). Resveratrol modulates epigenetic regulators of promoter histone methylation and acetylation that restores BRCA1, p53, p21CIP1 in human breast cancer cell lines. BioFactors.

[107] Izquierdo-Torres E., Hernández-Oliveras A., Meneses-Morales I., Rodríguez G., Fuentes-García G., Zarain-Herzberg A. (2019). Resveratrol up-regulates ATP2A3 gene expression in breast cancer cell lines through epigenetic mechanisms. Int. J. Biochem. Cell Boil..

[108] Liu X., Li H., Wu M.-L., Wu J., Sun Y., Zhang K.-L., Liu J. (2019). Resveratrol Reverses Retinoic Acid Resistance of Anaplastic Thyroid Cancer Cells via Demethylating CRABP2 Gene. Front. Endocrinol..

[109] Wang Z., Liu Y., Xue Y., Hu H., Ye J., Li X., Lu Z.-G., Meng F., Liang S. (2016). Berberine acts as a putative epigenetic modulator by affecting the histone code. Toxicol. Vitr..

[110] Koprowska K., Czyz M. (2010). Molecular mechanisms of parthenolide’s action: Old drug with a new face. Postepy Hig. Med. Dosw. (Online).

[111] Venturelli S., Berger A., Böcker A., Busch C., Weiland T., Noor S., Leischner C., Schleicher S., Mayer M., Weiss T.S. (2013). Resveratrol as a pan-HDAC inhibitor alters the acetylation status of histone [corrected] proteins in human-derived hepatoblastoma cells. PLoS ONE.

[112] Härmä V., Virtanen J., Mäkelä R., Happonen A., Mpindi J.-P., Knuuttila M., Kohonen P., Lötjönen J., Kallioniemi O.-P., Nees M. (2010). A Comprehensive Panel of Three-Dimensional Models for Studies of Prostate Cancer Growth, Invasion and Drug Responses. PLoS ONE.

[113] Mittler F., Obeïd P., Rulina A.V., Haguet V., Gidrol X., Maxim B. (2017). High-Content Monitoring of Drug Effects in a 3D Spheroid Model. Front. Oncol..

[114] Leach D., Buchanan G. (2017). Stromal Androgen Receptor in Prostate Cancer Development and Progression. Cancers.

